# Deciphering the Anti-Aflatoxinogenic Properties of Eugenol Using a Large-Scale q-PCR Approach

**DOI:** 10.3390/toxins8050123

**Published:** 2016-04-26

**Authors:** Isaura Caceres, Rhoda El Khoury, Ángel Medina, Yannick Lippi, Claire Naylies, Ali Atoui, André El Khoury, Isabelle P. Oswald, Jean-Denis Bailly, Olivier Puel

**Affiliations:** 1 Toxalim, Université de Toulouse, INRA, ENVT, INP Purpan, UPS, Toulouse, France; isauracaceres@hotmail.com (I.C.); rhodakhoury@gmail.com (R.E.K.); yannick.lippi@toulouse.inra.fr (Y.L.); claire.naylies@toulouse.inra.fr (C.N.); ioswald@toulouse.inra.fr (I.P.O.); olivier.puel@toulouse.inra.fr (O.P.); 2 Laboratoire de Mycologie et Sécurité des Aliments (LMSA), Département de Biochimie, Faculté des Sciences, Université Saint-Joseph, P.O. Box 11-514, Beirut 1107 2050, Lebanon; andre.khoury@usj.edu.lb; 3 Applied Mycology Group, School of Energy, Environment and AgriFood, Cranfield University, Cranfield MK43 0AL, Bedfordshire, UK; a.medinavaya@cranfield.ac.uk; 4 Laboratory of Microbiology, Department of Natural Sciences and Earth, Faculty of Sciences I, Lebanese University, Hadath Campus, P.O. Box 11-8281, Beirut, Lebanon; a.atoui@cnrs.edu.lb

**Keywords:** Aflatoxin B_1_, *Aspergillus flavus*, aflatoxinogenesis, molecular tool, gene regulation, eugenol

## Abstract

Produced by several species of *Aspergillus*, Aflatoxin B_1_ (AFB_1_) is a carcinogenic mycotoxin contaminating many crops worldwide. The utilization of fungicides is currently one of the most common methods; nevertheless, their use is not environmentally or economically sound. Thus, the use of natural compounds able to block aflatoxinogenesis could represent an alternative strategy to limit food and feed contamination. For instance, eugenol, a 4-allyl-2-methoxyphenol present in many essential oils, has been identified as an anti-aflatoxin molecule. However, its precise mechanism of action has yet to be clarified. The production of AFB_1_ is associated with the expression of a 70 kB cluster, and not less than 21 enzymatic reactions are necessary for its production. Based on former empirical data, a molecular tool composed of 60 genes targeting 27 genes of aflatoxin B_1_ cluster and 33 genes encoding the main regulatory factors potentially involved in its production, was developed. We showed that AFB_1_ inhibition in *Aspergillus flavus* following eugenol addition at 0.5 mM in a Malt Extract Agar (MEA) medium resulted in a complete inhibition of the expression of all but one gene of the AFB_1_ biosynthesis cluster. This transcriptomic effect followed a down-regulation of the complex composed by the two internal regulatory factors, AflR and AflS. This phenomenon was also influenced by an over-expression of *veA* and *mtfA*, two genes that are directly linked to AFB_1_ cluster regulation.

## 1. Introduction

Aflatoxin B_1_ (AFB_1_) is a mycotoxin produced by many fungal species belonging to the *Flavi* section of the *Aspergillus* genus, *A. flavus* being the most preoccupying species [1]. AFB_1_ is the most potent naturally occurring carcinogen [2], responsible for hepatocarcinoma; it is also an immunosuppressive agent and has been linked to growth impairment in children [3,4].

AFB_1_ is problematic in countries with tropical and sub-tropical climates where temperature and humidity conditions are optimal for fungal growth and toxin production [5]. However, this danger has spread beyond its predicted geographical borders and has reached countries previously considered as safe. In fact, in recent years, several surveys demonstrated the contamination of European crops by this toxin [6,7]. Fungal infection can occur at a pre- or post-harvest stage of cereal production, especially corn, but also oilseeds, nuts, spices and dried fruit [8].

Many strategies have been developed to reduce AFB_1_ contamination, either by preventing the fungal development or by blocking the toxin’s production after infection [9]. Natural extracts are considered as a possible alternative antimicrobial agent [10]. Indeed, organic and aqueous extracts from plants and spices, as well as essential oils, have demonstrated fungicidal and/or anti-toxinogenic properties [11,12,13]. For example, eugenol (4-allyl-2-methoxyphenol), the active compound of many anti-toxinogenic essential oils [14,15], blocks AFB_1_ production in aflatoxigenic fungi [16,17]. However, little is known about the molecular mechanism of this inhibition and the fungal pathways affected by eugenol remain to be determined.

AFB_1_’s biosynthetic pathway is well characterized and consists in *A. flavus*, of a cluster of 27 genes whose expression is governed by two internal regulators (AflR and AflS). AFB_1_’s biosynthesis is also interconnected with developmental genes that play a role in morphology, conidiation, or sclerotia formation [18,19], as well as genes encoding the velvet regulating proteins that coordinate primary and secondary metabolism (SM) [20]. Transcription factors (TFs) such as *mtfA* [21] and *fcr3* (*A. nidulans rsmA* orthologous) [22] and other TFs influenced by environmental factors like pH, nitrogen and carbon, can also interfere with aflatoxin production [23]. In the same way, genes related to oxidative stress regulation [24] as well as genes encoding cellular signal mediators such as *rasA*, [25] G-protein receptors [26] and oxylipins’ biosynthetic genes [27] influence toxin synthesis. This demonstrates the complexity of environmental signals and cellular pathways involved or interfering with mycotoxin production.

In the present study, a molecular tool was developed including genes directly implicated in aflatoxin production as well as genes involved in the upstream regulation of this toxin. This tool was used to determine the molecular mechanism of AFB_1_’s inhibition by eugenol. Hence, we demonstrate that this later compound acts at the transcriptomic level to inhibit AFB_1_ production by restricting the expression of its biosynthetic cluster. Moreover, we also reveal that this inhibition is mainly governed by the modification of *mtfA* and *veA’s* expression levels.

## 2. Results

### 2.1. Effect of Eugenol on Fungal Growth and Aflatoxin B_1_ Production

**Figure 1:**
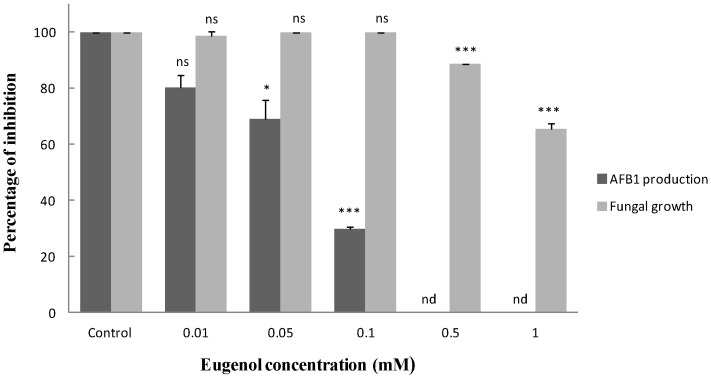
Effect of eugenol on Aflatoxin B_1_ (AFB_1_) production and fungal growth in *A. flavus* NRRL 62477. Results are expressed as percentage of the control value. AFB_1_ was estimated by High Performance Liquid Chromatography (HPLC) and fungal growth by colony diameter. Both measures were taken on day 4 on six biological replicates. ns = no significant changes; nd = not detectable; * *p*-value** < 0.05; *** *p*-value < 0.001.

Five different concentrations of eugenol were tested for their effect on both fungal development and AFB_1_ biosynthesis (Figure 1). Fungal growth was only slightly affected and colony diameter was reduced by 11.4% for 0.5 mM and 34.5% for 1 mM eugenol. By contrast, eugenol decreased AFB_1_ production in a dose-dependent manner with inhibitions of 19.8, 30.9, 70.2 and 100% at eugenol concentrations of 0.01, 0.05, 0.1 and 0.5–1 mM, respectively.

For subsequent assays, a concentration of 0.5 mM of eugenol was used allowing complete inhibition of AFB_1_ production with a limited impact on fungal growth. Since AFB_1_ production can be modulated by pH, this parameter was measured in the fungal cultures. Before incubation, pH values were 5.30 ± 0.06 and 5.23 ± 0.08 in control and treated cultures, respectively. After four days at 27 °C, both cultures displayed a mild but statistically significant (*p*-value = 0.049) acidification with pH means ± Standard Error of Mean (SEM) of 4.7 ± 0.03 for control and 4.4 ± 0.2 for eugenol treated cultures.

### 2.2. Effect of Eugenol on Aflatoxin Biosynthetic Pathway

**Figure 2:**
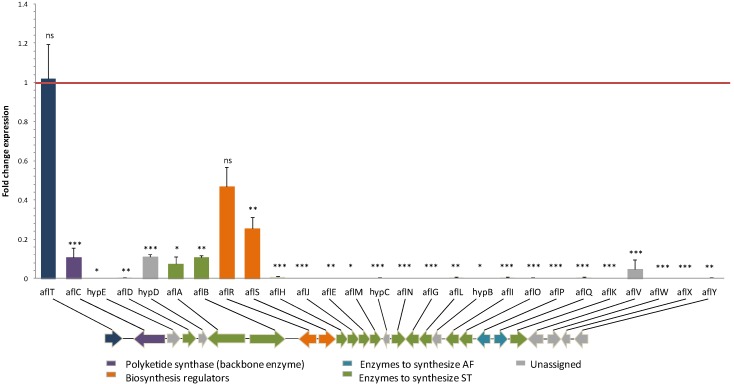
Fold change expression of genes belonging to the cluster responsible for aflatoxin biosynthesis in response to eugenol at 0.5 mM. Red line represents control expression level. Gene cluster organization was adapted from Amaike and Keller [1]; ns = no significant changes; * *p*-value < 0.05; ** *p*-value < 0.01; *** *p*-value < 0.001.

In *A. flavus*, AFB_1_’s biosynthetic pathway consists of 27 genes regrouped in a cluster where *aflR* and *aflS* are the two internal regulators. Following the addition of 0.5 mM eugenol, AFB_1_ was completely inhibited and all cluster genes with the exception of *aflT* (*p*-value *=* 0.8667) were strongly down-regulated. In fact, the expression of 19 out of 27 genes was almost completely inhibited, whereas five others had 10- to 20-fold reductions in expression levels compared to control conditions (Figure 2). Therefore, the extent of down-regulation was mildly different according to the chronological intervention level of the encoded enzyme in the biosynthetic pathway.

As an illustration, fundamental genes involved in the first steps of AFB_1_’s enzymatic cascade, such as *aflC*, encoding the polyketide synthase A, and two of fatty acid synthase genes aflA (fas-2) and aflB (fas-1), appeared less affected by eugenol compared to further intermediate genes such as *aflO (omtB)*, *aflP (omtA)* and *aflQ (ordA)*, the latter being an enzyme in charge of the final transformation of AFB_1_. For those genes, expression was almost completely inhibited by eugenol (*p*-values < 0.0001).

The reduced expression of cluster genes went with a decreased expression of the internal regulators. Concerning *aflS,* it saw a 3.9-fold down-regulation (*p*-value = 0.0030) whereas *aflR*’s expression level was not significantly affected by eugenol addition (*p*-value = 0.0522), although a diminution averaging at half was observed.

### 2.3. Effect of Eugenol on Regulatory Factors Linked to AFB_1_ Production

**Figure 3:**
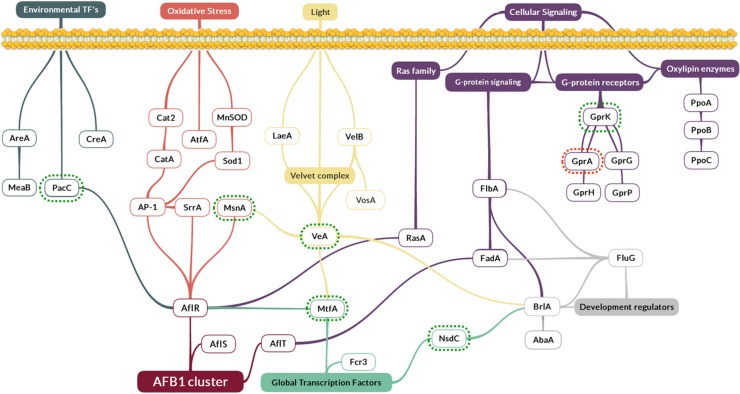
Schematic representation of the 33 selected regulatory factors linked to AFB_1_ cluster in *A. flavus*. This hypothetical schema represents a simplified version of the different interactions between the regulatory factors and Aflatoxin‘s cluster. Schema was constructed based on gene interaction data described by the following works: [10,19,20,21,22,23,24,26,28,29,30,31,32,33,34,35,36,37,38]. Up- or down-regulation of genes upon eugenol addition is represented by green an red dotted lines, respectively.

Since AFB_1_ biosynthesis is strongly interconnected with several other fungal biosynthetic pathways, a large number of genes considered as regulatory factors were analyzed. They included the velvet complex, genes involved in oxidative stress response, environmental and global transcription factors, genes involved in cellular signaling (oxylipins, Ras family and G-protein signaling and receptors) and developmental regulators (Figure 3).

**Figure 4:**
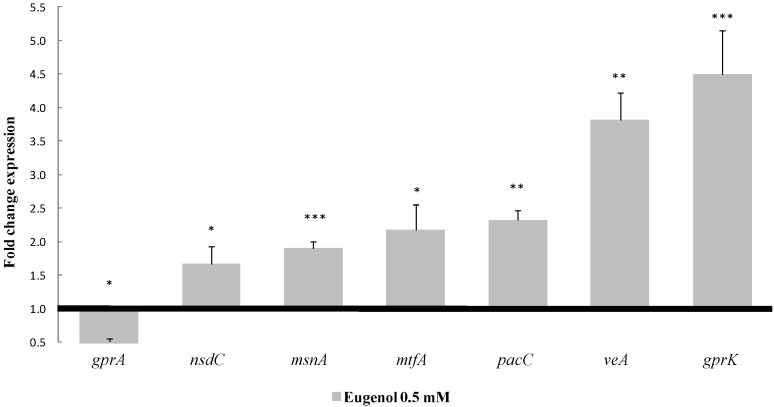
Fold change levels of the seven affected regulatory factors in presence of eugenol at 0.5 mM. Baseline represents control expression level; * *p*-value < 0.05; ** *p*-value < 0.01; *** *p*-value < 0.001.

As shown in Figure 4, among the 33 tested genes, only seven presented significant modifications of their level of expression upon eugenol exposure.

These genes were:

- The global regulator gene *veA*, belonging to the velvet complex. It was over-expressed with a 3.8-fold change compared to the control (*p*-value = 0.002);
- *mtfA*, a putative C_2_H_2_ zinc finger transcription factor. It presented the same up-regulated pattern, increasing its expression by 2.2 times (*p*-value = 0.0297);
- *nsdC,* of the global transcription factors, whose expression was increased by 1.7 times (*p*-value = 0.0100);
- *gprK,* which was the most affected gene among the five G-protein coupled receptors analyzed here. This gene was over-expressed by 4.5 times (*p*-value = 0.0009). By contrast, *gprA* was down-regulated by 0.45 times (*p*-value = 0.0177);
- The *msnA* gene was increased by 1.9 times (*p*-value < 0.0001), whereas no significant changes were observed for other genes implicated in the oxidative stress response such as superoxidase dismutases, catalases or oxylipins;
- Finally, *pacC*’s expression, a zinc finger transcription factor related to pH, was increased by 2.3 times (*p*-value = 0.0098).

## 3. Discussion

A number of natural extracts were identified as being able to down-modulate the synthesis of the carcinogenic mycotoxin AFB_1_ in *A. flavus* [39]. The use of such inhibiting compounds could therefore represent an alternative strategy to the use of pesticides to control crop contamination. However, to date, the precise molecular mechanism responsible for this effect is only poorly documented. Indeed, mycotoxin production is a complex phenomenon based on the presence of biosynthetic clusters in toxigenic fungi whose regulation is governed by many environmental and physiological processes.

In order to better understand the inhibition of AFB_1_ production by such natural extracts, we developed a molecular tool allowing the simultaneous analysis of the expression of both the AFB_1_ cluster and a large number of global regulatory genes involved in different cellular pathways. We used it to characterize the mechanism of action of eugenol, a compound present in many essential oils and that has been previously identified as an AFB_1_ inhibitor. Eugenol has been extensively studied for its many biological effects including antimicrobial, anti-inflammatory, anti-oxidant and anticancer activities. However, to date, no precise cellular target was identified even though the interaction with cell membrane may represent a key point in the biological effects of this molecule [40].

### 3.1. Eugenol Inhibits the Expression of Aflatoxin Cluster Genes in A. flavus

The inhibition of AFB_1_ production by a toxigenic strain of *A. flavus* following eugenol addition is accompanied by the down-regulation of all but one aflatoxin cluster genes.

We demonstrated that the expression of 19 out of 27 genes of the cluster was almost no more detectable after eugenol exposure, whereas the others saw 10- to 20-fold reductions in their expression levels. These results are coherent with a very recent work of Jahanshiri *et al.* [16] showing that eugenol decreased the expression of some of Aflatoxins (AF)’s cluster genes in *A. parasiticus.* In that previous study, only five genes of the pathway were analyzed. Our present work extended this finding to the whole genes involved in the biosynthesis of AFB_1_.

AFB_1_’s cluster is internally regulated by two genes, *aflR* and *aflS*, whose activation is governed, independently one from another, by external regulators. An interaction between AflR and AflS is reportedly required for aflatoxinogenesis [41]. Kong *et al.* [42] reported that the activation by these two proteins led to the formation of a functional activation complex in the proper ratio of four AflS to one AflR*.* In our study, the expression of *aflR* was not significantly decreased in cultures exposed to eugenol, although a major down-regulation tendency was observed. Such a finding was already described in several studies where even low-level changes of *aflR*’s expression levels were accompanied by a severe decrease of structural genes [25,43,44]. However, levels of *aflS* transcripts were significantly reduced, which might have led to an alteration of the ratio between AflR and AflS and thus the formation of a limited number of active complexes. Consequently, AflR-binding sites were not attained and the transcription of the cluster was not activated (Figure 2). All of the cluster genes regulated by *aflR* had their expression levels severely decreased by eugenol’s addition. The genes intervening in the later stages of AFB_1_’s enzymatic cascade (*aflM*, *aflN*, *aflX*, *aflO*, *aflP*, *aflQ* and *hypB*) were more impacted than those involved in the beginning stages (*aflA*, *aflB*, *aflC*) leading to the polyketide structure [45]. The limited AflR/AflS complexes formed might have been promptly used up at the beginning of AFB_1_’s synthesis and were no longer sufficiently available for the proper activation of the rest of the cluster genes.

For *aflT*, the expression levels were not significantly different between control and eugenol-treated cultures. In fact, *aflT*, a Major Facilitator Superfamily (MFS) transporter encoding gene, is regulated neither by AflR nor by its co-activator AflS, due to the absence of an AflR binding-site on its promoter, but rather by the FadA-dependent G-protein signaling pathway [46]. Moreover, the expression of none of the genes belonging or affected by the latter pathway, notably *fadA*, *flbA*, *fluG* and *brlA* [34], has been altered by eugenol’s addition (Table S1).

### 3.2. Eugenol Alters the Expression of Global Regulation Factors

Eugenol‘s transcriptomic effect goes upstream of AF’s cluster genes, affecting genes encoding general transcriptional regulating factors.

#### 3.2.1. The Pivotal Role of MtfA, VeA and MsnA in Eugenol*’*s Molecular Mechanism

*MtfA* is a global transcription factor, regulating sterigmatocystin/aflatoxin biosynthesis as well as other secondary metabolites clusters [31]. The deletion as well as the over-expression of *mtfA* has been shown to inhibit the expression of *aflR* and subsequent sterigmatocystin production in *A. nidulans* [21]*.* Also, in the recent work of Zhuang *et al.* [47] on peanut seeds infected with Δ*mtfA* and over-expressed (OE) *mtfA* gene on *A. flavus* strains, it was observed that the decrease in AFB_1_ production was greater when there was a 2.75-fold *mtfA* over-expression compared to the wide-type strain, than deleted, and went with a decreased expression of *aflR*. Although the *mtfA* gene is over-expressed at a similar level as in the above-quoted study [47], this does not mean that MtfA is the direct molecular target of eugenol. A kinetic study of gene expression, using our molecular tool coupled to AF production analysis, could be used in order to determine the time course evolution of modulated genes.

Furthermore, *mtfA’*s expression is highly dependent on that of *veA*. As discussed in the study by Lind *et al.* [31], MtfA interacts with VeA in *A. nidulans* and the expression of *mtfA* (AN8741.2) was decreased by 5 times in an *A. nidulans* Δ*veA*. All of the above findings are in favor of the notion that the over-expression of *mtfA* in *A. flavus* is a result of the increased expression of *veA* upon eugenol addition. The interaction of these two regulators could then be responsible for *aflR’*s down-regulation and the succeeding inhibition of AF*’*s biosynthetic pathway in *A. flavus*. Moreover, VeA is by itself essential for the transcription of both *aflR* and *aflS* and, consequently, the production of aflatoxins [48]. This global transcription regulator plays a key role in secondary metabolite production [49]. Depending on its abundance in the cell, it may act as a repressor or as an activator [50]. For instance, the expression of fumagillin and fumitremorgin G gene clusters was inhibited in a overexpressing *veA* (OE:*veA*) strain of *A. fumigatus* [51]. Therefore, an over-expression of *veA* (3.8-fold) observed here is compatible with the down-regulation of *aflR* and *aflS* and the subsequent inhibition of aflatoxin production in eugenol-treated cultures. Furthermore, as in many filamentous fungi, SM, and aflatoxin production in particular are often induced as a response to Reactive Oxygen Species (ROS) formation [52]. It has been also demonstrated that VeA plays a critical role in protecting *A. flavus* from oxidative stress. VeA positively regulates the expression of oxidative stress tolerance genes such as *msnA* in *A. flavus* [38] and also in *A. nidulans* [31]*.* Conversely, levels of ROS as well as AFB_1_ increased in Δ*msnA* *A. flavus* and *A. parasiticus* strains [53]. Furthermore, it has been demonstrated that aflatoxin biosynthesis is itself a source of intracellular ROS, and the over-expression of *msnA* could then also be the outcome of AFB_1_*’*s inhibition [54].

Accordingly, an oxidative-stress alleviating condition, such as the addition of eugenol, could lead to an increased expression of *veA* and consequently of *msnA*. In our study, *msnA* transcripts increased by 1.9 times along with AFB_1_*’*s inhibition in eugenol-treated cultures, following *veA’*s over-expression. This effect is possibly linked to a decrease of intracellular ROS.

#### 3.2.2. The Putative Implication of Other Regulatory Factors and Signaling Proteins

Eugenol addition also alters the expression of other transcription-regulating factors such as (i) *nsdC* and (ii) *pacC* encoding a pH-dependent transcription factor, as well as (iii) the *grpA* and *grpK* genes involved in the G-protein signaling pathway. Except for *grpK*, these genes have been shown to be closely related to the expression of AF cluster genes and AFB_1_ production [19,26,55,56]. However, to date, the exact mechanism of this interaction has to be clarified.

## 4. Conclusions

In this study, we bring out the transcriptomic inhibition behind eugenol*’*s AFB_1_-repressing action. This is the first time, to our knowledge, that the expression of all the 27 genes involved in AFB_1_ synthesis has been studied on an inhibitor molecule. In the presence of eugenol, AFB_1_ cluster genes were strongly down-modulated following decreased expression of its regulating complex AflR/AflS. We also demonstrated that this went with a modulation of seven regulatory factors. We then highlighted the involvement of *mtfA*, *veA* and *msnA* in this inhibition.

## 5. Materials and Methods

### 5.1. Chemicals and Reagents

Aflatoxin B_1_ and eugenol standards were purchased from Sigma-Aldrich (Saint-Quentin-Fallavier, France) and dissolved in methanol and ethanol, respectively. Stock solutions were stored at 4 °C until use. All analytical grade solvents were purchased from Thermo Fisher Scientific (Illkirch, France).

### 5.2. Fungal Strain and Culture Conditions

The *Aspergillus flavus* strain NRRL 62477 used in this study [57] was maintened in the dark on a Malt Extract Agar (MEA) medium (Biokar Diagnostics, Allone, France) at 27 °C.

For experiments, 10 μL of a calibrated spore suspension (10^6^ spores/mL) prepared from a seven-day culture was used to centrally inoculate the MEA medium covered with sterile cellophane layers (Hutchinson, Chalette-sur-Loing, France) as described by Leite *et al.* [58]. Firstly, five different concentrations of eugenol (0.1 to 1 mM) were tested to determine the concentration able to inhibit AFB_1_ with a limited impact on fungal development, as measured by colony diameter. The concentration of 0.5 mM was selected for further experiments. Eugenol dilutions were prepared to add only 20 μL of ethanol in the culture medium, this concentration having been identified as a no-effect dose on both fungal growth and AFB_1_ production. Control cultures were performed by adding only 20 μL of ethanol in the medium. Six replicates of each group were prepared and incubated for four days at 27 °C in the dark. At least three replications of the experiment were performed. The pH of all media was taken before and after incubation using a food pH-meter H199161 (Hanna Instruments, Tanneries, France).

### 5.3. Aflatoxin B_1_ Extraction and Determination by HPLC

For AFB_1_ extraction, culture media were mixed with 25 mL of chloroform. Samples were agitated for 2 h on a horizontal shaking table at 160 rpm at room temperature. Chloroform extract was filtered through a Whatman 1PS phase separator (GE Healthcare Life Sciences, Vélizy-Villacoublay, France), evaporated at 60 °C until dry and dissolved in 500 μL of a water-acetonitrile-methanol (65:17.5:17.5; *v*/*v*/*v*) mixture. To eliminate possible impurities, all samples were filtered through a 0.45 μm disk filters (Thermo Scientific Fisher, Villebon-Sur-Yvette, France). The analysis of samples was done with a Dionex Ultimate 3000 UHPLC system (Thermo Scientific, Illkirch, France) using a liquid chromatography column, Luna^®^ C18 (125 × 2 mm, 5 μm, 100 Å) (Phenomenex, Torrance, CA, USA) at 30 °C. Separation conditions were adapted from Fu *et al.* [59] with mild modifications. A 20 min isocratic mode was delivered at 82.5% of eluent A: acidified water (0.2% of acetic acid) and acetonitrile (79:21 *v*/*v*); and 17.5% of eluent B: pure methanol. A flow rate of 0.2 mL/min was used and 10 μL of extract was injected. AFB_1_ was detected by a fluorescent detector at 365/430 nm excitation/emission wavelengths. Peak identity was confirmed by analyzing absorption spectrum with a diode array detector coupled to the system. Production levels of AFB_1_ on media were calculated based on a standard calibration curve.

### 5.4. Isolation of Fungal RNA and Reverse Transcriptase-Polymerase Chain Reaction (RT-PCR)

At the end of incubation, mycelia were separated from the medium and ground up under liquid nitrogen. The RNA was purified as recommended by the manufacturer from 100 mg of mycelium using a Qiagen RNeasy PlusMinikit (Qiagen, Hilden, Germany) and including a gDNA eliminator column. The quality of RNA was verified by gel electrophoresis (1.2% agarose) and concentrations were measured using a NanoDrop ND1000 (Labtech, Palaiseau, France). The A_260_/A_280_ ratio was measured [60], and each sample was adjusted to a final RNA concentration of 300 ng/μL.

First-strand cDNA synthesis was carried out by RT-PCR. Reverse transcription took place in a 20 μL reaction mixture containing 10 μL of RNA, 200 U of RevertAid Reverse transcriptase, 4 μL of 5× Reaction Buffer, 20 U of RNase inhibitor (Thermo Scientific, Illkirch, France), 2 μL of 10 mM dNTP (Euromedex, Souffelweyersheim, France), 1 μL of sterile water, and 1 μL of oligo (dT) Bys 3' Primer: (5'-GCTGTCAACGATACGCTATAACGGCATGACAGTGTTTTTTTTTTTTTTT-3').

A first denaturation was done at 70 °C for 5 min and reverse transcription was performed as follows: 5 min at 37 °C; 60 min at 42 °C and 15 min at 85 °C.

### 5.5. Design and Validation of q-PCR Primers

All primer sets were designed based on the genomic data of the *Aspergillus flavus* strain NRRL3357 (GenBank accession number EQ963478A). A total of 62 genes primer pairs were designed including all 27 AFB_1_ cluster genes. It must be noted that *aflF* and *aflU* were not followed in this study, as the promoter regions of both genes are missing in *A. flavus* species, thus their inability to produce type-G aflatoxins. Other regulatory factors directly or indirectly related to AFB_1_’s production (Figure 3) were included, along with two housekeeping genes, *b*-*tubulin* and *gpdA*. All primer pairs were designed to amplify a 50–150 bp fragment based solely on the coding sequence of the corresponding genes, with at least one of the primers extending on an exon/exon junction in order to avoid undesirable genomic DNA amplification. Primer-dimer or self-complementarities were evaluated using the PrimerExpress 2.0 software (Applied Biosystems, Courtaboeuf, France). All primers were synthesized by Sigma Aldrich (Saint-Quentin Fallavier, France) and tested with four different concentrations (300/300; 300/900; 900/300 and 900/900 nM) after reception to determine their optimal concentrations in the mix. Primer validation was carried out with the best amplification curve and dissociation curves were used to confirm the good amplification of each gene. At least three biological replicates of *A. flavus* NRRL62477 were used to validate the amplification specificity. Negative controls in which no reverse transcriptase enzyme was added and a no template control were included to control reagents contamination. Primer sequences and their concentrations are listed in Table S2.

### 5.6. Analysis of the Expression of the Genes Linked to Aflatoxin B_1_ Biosynthesis

Experiments were carried out using a ViiA7 Real-Time PCR System (Applied Biosystems, Forster City, CA, USA). The 384 well-plates were prepared by an Agilent Bravo Automated Liquid Handling Platform (Agilent Technologies, Santa Clara, CA, USA). Each well contained a final volume of a 5 μL mix: 2.5 μL of Power SYBR^®^ Green PCR Master Mix (Applied Biosystems, Warrington, UK) used as a fluorescent dye, 1.5 μL of each primer set and 1 μL of cDNA material. Three-step quantitative PCRs were performed as follows: a first one-hold stage at 95 °C for 10 min followed by 45 cycles (95 °C for 15 s and 60 °C for 30 s), and a final extending step (95 °C for 15 s, 60 °C for 1 min and 95 °C for 15 s) for melt curve analysis. The results were analyzed with a Quant-Studio Real time PCR software v1.1 (Applied Biosystems, Courtaboeuf, France). Housekeeping genes were analyzed with Normfinder algorithm [61] and the more stable was used as a reference for normalization in the 2^−ΔΔC*t*^ analysis method [62]. Five distinct experiments were done, each including at least three biological replicates of each condition.

### 5.7. Statistics

Student*’*s *t*-test was used to analyze the differences between control and treated samples. The differences were considered to be statistically significant when the *p*-value was lower than 0.05.

## Supplementary Materials

The following are available online at www.mdpi.com/2072-6651/8/5/123/s1, Table S1: Gene expression values of regulatory factors that were not impacted upon Eugenol addition. Values are expressed in fold change and compared to a control fixed at 1; Table S2: Primer sequences of the 62 designed genes.

## Abbreviations

*A. flavus*	*Aspergillus flavus*
AF	Aflatoxins
AFB_1_	Aflatoxin B_1_
GPCRs	G-protein coupled receptors
HPLC	High Performance Liquid Chromatography
MEA	Malt Extract Agar
MFS	Major Facilitator Superfamily
mM	Millimolar
nd	Not detectable
ns	No significant changes
OE	Over-expresssed
ROS	Reactive oxygen species
SEM	Standard Error of Mean
SM	Secondary metabolism
TFs	Transcription factors
