# Supplementary material for: Deciphering the Anti-Aflatoxinogenic Properties of Eugenol Using a Large-Scale q-PCR Approach
Source: Toxins (Basel). 2016 Apr 26;8(5):123. doi: 10.3390/toxins8050123 (PMC4885038; doi:10.3390/toxins8050123)
Supplement: Supplementary file 1 [file toxins-08-00123-s001.pdf]

Supplementary Materials: Deciphering the Anti-Aflatoxinogenic Properties of Eugenol Using a Large-Scale q-PCR Approach

Isaura Caceres, Rhoda El Khoury, Ángel Medina, Yannick Lippi, Claire Naylies, Ali Atoui, André El Khoury, Isabelle P. Oswald, Jean-Denis Bailly and Olivier Puel

Table S1. Gene expression values of regulatory factors that were not impacted upon Eugenol addition. Values are expressed in fold change and compared to a control fixed at 1.

Gene Name	Values	*p*-Value
abaA	1.13	0.8179
ap-1	1.12	0.4589
areA	1.29	0.4578
atfA	2.09	0.0537
brlA	0.77	0.1953
cat2	1.13	0.8072
catA	1.84	0.3623
creA	1.32	0.648
fadA	0.95	0.891
fcr3	0.73	0.0836
flbA	0.55	0.2958
fluG	1.11	0.5914
gprG	1.52	0.1591
gprH	0.84	0.3971
gprP	1.29	0.2462
laeA	1.05	0.7673
meaB	1.43	0.1367
mnSOD	0.84	0.3045
ppoA	1.26	0.2914
ppoB	0.77	0.4413
ppoC	0.89	0.6097
rasA	0.88	0.4225
SOD1	0.95	0.8985
srrA	1.69	0.2001
velB	1.43	0.3543
vosA	1.49	0.1905

Table S2. Primer sequences of the 62 designed genes.

Access Number	Gene	nM	Primers Sequences 5′ to 3′	
AFLA_139380	*aflA*	*300/900*	F: CCAGTCGGTGGTGTGCAAAA	R: CGCAGCACCCCAGAGCTT
AFLA_139370	*aflB*	*300/300*	F: GTCTGCGCAGGCCATTTC	R: AGCTCTTGGCCTTCAACAGTCTC
AFLA_139410	*aflC*	*300/300*	F: CTGACCCCGGCATTTCG	R: TGCCAGATTCCTCATATTCCCG
AFLA_139390	*aflD*	*300/900*	F: AGGCATCTGTGCTCGGATTG	R:TGCCCCGATGTAGTCTCCTTAGT
AFLA_139310	*aflE*	*900/300*	F: CTGCAACACTCGGCTAGAAACA	R: GAAGGGTTCGATCGGCTGT
AFLA_139260	*aflG*	*900/900*	F:CATGCAGAGTAACCGCATCGT	R: TTGTAGACCGACGGATGTCCC
AFLA_139330	*aflH*	*900/900*	F: CCAGCTCCACGGTCGG	R: CTTAAGGTCAAAGATTCCCTCGG
AFLA_139230	*aflI*	300/300	F: GATCGGCTCGTTTGAGGGA	R: GCAAAAATGATATTCAGCTGGTTTGAC
AFLA_139320	*aflJ*	*300/900*	F: GCTCCGTGGCACCAGTT	R: TCGTCACGCTCTCATCGG
AFLA_139190	*aflK*	*300/300*	F: ACAGCGCGAAATGTTTTATATGC	R:GCGCCTTTCGAGCCTCTG
AFLA_139250	*aflL*	*300/300*	F: CCGATTCGTACCCGAAGAGAC	R: TCGGCAAAGTAGGTACCGAGAT
AFLA_139300	*aflM*	*900/900*	F: TCCTGGAGCCATTAAGACTGATATGT	R: GCCAAGCGGCGCACTC
AFLA_139280	*aflN*	*300/300*	F: CCAGGCTCGCCAGGAAGT	R: AGCTTGAACTCTGGCATAGGAAGG
AFLA_139220	*aflO*	*300/900*	F: CCCCAAGAGTATACCTCGAGTGC	R: AAGGTCCCGAGATGTCGAATAGTT
AFLA_139210	*aflP*	*900/300*	F: TATTCTACATGACTATCCCGATGCTG	R: GCGCGACTTGCTTGGGT
AFLA_139200	*aflQ*	*900/900*	F: GGGAGGATCGGACACGACA	R: CATGGCCACGAAAAAGCTAGAC
AFLA_139360	*aflR*	*900/900*	F: GCGGCACAGCTTGTTCTGA	R: CCGGTATCCCTGCTGCATC
AFLA_139340	*aflS*	*900/900*	F: AACGGTCGTGCATGTGGG	R: CGGCCTTAGCTTCTGTCTGC
AFLA_139420	*aflT*	*300/300*	F: GTAGCATGCGCCTCCGTCT	R: GCGAATGGCTTGGAACCAG
AFLA_139180	*aflV*	*300/900*	F: CCTGGTGGTTCCTCGCTGT	R: TGAAATATGCGGTTTGAAGAATCTTG
AFLA_139170	*aflW*	*300/900*	F: CGATGTCTTTGTGCGGACG	R: GTGTTGCCCGCTAGCACTC
AFLA_139160	*aflX*	*900/900*	F: GATCCACGCCTACTGCCGT	R: GATCTCAGGATGCAATCGGTTC
AFLA_139150	*aflY*	*300/300*	F: GGTGTCCCTACGGTCTTAAATGAGT	R: CAATGGGTGGAGAAAGCCAGA
AFLA_139240	*hypB*	*900/300*	F: TCCTGCACTTCAAGCTACCGC	R: GACGCTCATCATGGCTCCG
AFLA_139400	*hypC*	*900/900*	F: GGTCCTTTTTGACGGGAGCC	R: TCTCGATAAGAATGGGAATGGTGA
AFLA_139270	*hypD*	*300/900*	F: TTGCGTTTGCCGACGG	R: GCGCATAACTCCAATGTCCC
AFLA_139290	*hypE*	*900/900*	F: AAGTTCCCGACTTTCCCGC	R:CAAAGTTGATAATAGCAAATCCGTCATA
AFLA_029620	*abaA*	*900/900*	F: TACAAAACTACTGGCAAAAGGAGGTC	R: CCCGTACAAGCCTCTCCCA
AFLA_129340	*ap-1*	*900/300*	F: CTTGCACCAAGATTTGGGACC	R: TCGTACGCAATTCGGAGCA
AFLA_049870	*areA*	*300/900*	F: CAGCCAATCCGGCGAAC	R: CCCATTCCTGACTAGCCCCTT
AFLA_031340	*atfA*	*900/900*	F: GCAGTTACAGGCACCTTAACACATC	R: CGGCGCCTGGGAGTCTT
AFLA_082850	*brlA*	*300/300*	F: GCGTGCCTTCTCCCGC	R: GGAGTCCATCCGGTGTGAGTT
AFLA_068620	*β-tub*	*300/900*	F: AACGTCTACTTCAACGAGGCCA	R: GTACCAGGCTCAAGATCAACGAG
AFLA_122110	*cat2*	*300/900*	F: TGGCAGCAGTGACGGAAAG	R: AGCCCAAGCGGCAACAA

Table S2. *Cont.*

AFLA_056170	*catA*	*300/900*	F: ATTAAGTTCCCGGATTTTGTCCA	R: GGTACCTCGTTGTGCGGCT
AFLA_134680	*creA*	*300/900*	F: CAGCCGGAAGGCCCC	R: CATATCTTGCACCGCGACCT
AFLA_018340	*fadA*	*900/900*	F: TACATCCTCAACCGCTTCGTG	R: GGTTCTCCTGGATGATGATGTCATT
AFLA_133560	*fcr3*	*300/900*	F: GGCACAGAACAGAGCAGCTCAA	R: GCACATGTCGCTCCTTTCGTT
AFLA_134030	*flbA*	*300/900*	F: CTGGCTGCAGCATATGGTCTT	R: CATTGATTCGTCATCCCCTACAG
AFLA_101920	*fluG*	*300/300*	F: CGCCGGTTACCTAGGCGT	R: TTGGCGGCATCGTGGA
AFLA_025100	*gpdA*	*900/900*	F: CGTGTTGTTGACCTCATTGCCT	R: GGTGACCTGATAATCCGGGAAC
AFLA_060740	*gprA*	*900/900*	F: CTTCTGCAGTATCTTCATTATCAAGCTC	R: AATCGAAAGCAGCGCTGG
AFLA_067770	*gprG*	*300/900*	F: CCGCATTCAGCGGAAACTT	R: GGCGTTAGGGTTTGTGAGGAG
AFLA_006920	*gprH*	*300/900*	F: TTGATTGTCACCTGGGTTCCTTC	R: CATAGTTCAGAGCAAAGTTGTGGGA
AFLA_009790	*gprK*	*300/900*	F: CTTGCCATGCAGGTAGTCAAC	R: TCCAAATGGACAGGCAAATCC
AFLA_088190	*gprP*	*300/300*	F: CGATGCAGTCTTTCTAAGGGTGG	R: GCCATCGATTGTACCATGTGTGA
AFLA_033290	*laeA*	*900/900*	F: TGCCAACAAATACCCAGACG	R: TCGCAATTCTTTGGGTGGTT
AFLA_031790	*meaB*	*900/900*	F: CCAAAAGCACTATGATCAGCTTGAA	R: CCCACGGACGACTCATGC
AFLA_033420	*mnSOD*	*300/300*	F: GGTGGCTAATGGACCGGG	R: CATACGAGGCCTTGTTATTGAAGTACTG
AFLA_110650	*msnA*	*900/900*	F: GACGTCCATGCCGAGCTTT	R: AATTGGCGTTGATGAAGACAGG
AFLA_091490	*mtfA*	*300/900*	F: GCATCGACATATGCTGCAAAG	R: GGCGCAAAGGTGGAGTAGG
AFLA_131330	*nsdC*	*300/900*	F: TCCAGGACCACATGACTCCAA	R: GCTGTGCCCTTAAAGCAGCTACT
AFLA_030580	*pacC*	*900/300*	F: CAAGGATGTTCTGAGAAGTGTCCC	R: CGTTCGCAGACGTGCTCATA
AFLA_026790	*ppoA*	*300/900*	F: ATCAAGAGGCTGGCGAAGGT	R: GGGCGCCTTCCATGAAGTA
AFLA_120760	*ppoB*	*300/900*	F: AGTCTCTATGCGTTCCCTATGACTTG	R: CGCTCCCTTCCCGGAAT
AFLA_030430	*ppoC*	*300/300*	F: GTTCACAATCAGGCTCAAATGTTC	R: CAGGCAGTAGCGCATCAACTT
AFLA_132380	*rasA*	*300/300*	F: GCGAGCCGTCACAGTAGAAGAG	R: CGGATGTTTCGATAAATTTGCAG
AFLA_099000	*sod1*	*900/300*	F: CGAGAGCGTACTTGGCCGT	R: TCTTCTTGGACTCCGGGTGTT
AFLA_034540	*srrA*	*300/900*	F: TGTCGTCAGATTGGCGGAA	R: GCCTCGAGTCCGTCGAAAG
AFLA_066460	*veA*	*300/300*	F: CGTCAGCCGGATCACTCG	R: GACGGTCCGCAGAGGACTT
AFLA_081490	*velB*	*300/300*	F: GGCTTCGGCGACAAGGAT	R: TCTGCGTTCCACAAGTCTACCA
AFLA_026900	*vosA*	*300/900*	F: GGGTGTTCCAGATTATGCGATG	R: CCCGTTGAATCCCCCGTAG
